# New leads for fragment-based design of rhenium/technetium radiopharmaceutical agents

**DOI:** 10.1107/S2052252517003475

**Published:** 2017-04-11

**Authors:** Alice Brink, John R. Helliwell

**Affiliations:** a Department of Chemistry, University of the Free State, Nelson Mandela Drive, Bloemfontein, Free State 9301, South Africa; b School of Chemistry, University of Manchester, Brunswick Street, Manchester M13 9PL, England

**Keywords:** rhenium, technetium, radiopharmaceutical agents, fragment-based design, two X-ray wavelengths

## Abstract

Multiple possibilities for the coordination of *fac*-[Re(CO)_3_(H_2_O)_3_]^+^ to a protein have been determined and include binding to Asp, Glu, Arg and His amino-acid residues as well as to the C-terminal carboxylate in the vicinity of Leu and Pro. The large number of rhenium metal complex binding sites that have been identified on specific residues thereby allow increased target identification for the design of future radiopharmaceuticals. The core experimental concept involved the use of state-of-art tuneable synchrotron radiation at the Diamond Light Source to optimize the rhenium anomalous dispersion signal to a large value (*f*′′ of 12.1 electrons) at its *L*_I_ absorption edge with a selected X-ray wavelength of 0.9763 Å. At the Cu *K*α X-ray wavelength (1.5418 Å) the *f*′′ for rhenium is 5.9 electrons. The expected peak-height increase owing to the optimization of the Re *f*′′ was therefore 2.1. This X-ray wavelength tuning methodology thereby showed the lower occupancy rhenium binding sites as well as the occupancies of the higher occupancy rhenium binding sites.

## Abstract 2

The identification of multiple amino-acid coordination sites which can bind to the radiopharmaceutical synthon *fac*-[Re(CO)_3_]^+^ is reported.

## 1. Introduction   

Rhenium-188/186 and technetium-99m tricarbonyl complexes have shown much potential as therapeutic and diagnostic radiopharmaceuticals. The synthetic kit of the *fac*-[*M*(CO)_3_]^+^ [where *M* is rhenium(I) or technetium(I)] core, based in water, makes this starting synthon a highly attractive pharmaceutical model which coordinates to multiple ligands (Alberto *et al.*, 1999 ▸; Schibli *et al.*, 2000 ▸). We report here the coordination of multiple *fac*-[Re(CO)_3_]^+^ complex fragments to a protein that was selected to provide a full range of exposed amino acids. Both monodentate and bidentate complex coordination were observed to the aspartic acid, glutamic acid, arginine, leucine, proline and histidine residues, indicating preferential binding to neutral and anionic N and O atom donors with p*K*_a_ values varying from 3.71 to 4.15 (Cantor & Schimmel, 1980 ▸; Betts & Russell, 2003 ▸). These structural observations are critical for the development of radiopharmaceutical drug design using the *fac*-[*M*(CO)_3_]^+^ (where *M* is technetium-99m, rhenium-186 or rhenium-188) radionuclide core (Alberto *et al.*, 1995 ▸, 1998 ▸, 2001 ▸), in particular considering the fragment-based drug-design (FBDD) method (Joseph-McCarthy *et al.*, 2014 ▸; Erlanson, 2012 ▸; Murray *et al.*, 2012 ▸), as specific protein–ligand binding of low-molecular-weight fragments can now be exploited to derive a model for possible drug-like lead compounds.

The recent crystal structure studies of cisplatin and carboplatin, chemically transformed into iodoplatin, gave new leads for radiation therapy and emphasized the importance of three-dimensional structural knowledge in new compound discovery, as an alternative treatment in tumour radiation therapy was suggested based on the X-ray crystal structure analyses (Tanley & Helliwell, 2014 ▸). In this new study, we provide the three-dimensional information required for new drug-lead development for rhenium-compound interactions with a wide range of amino acids, which was derived from X-ray crystallography supplemented with extensive analysis of the Cambridge Structural Database (CSD; Allen, 2002 ▸) and was supported by the precise distance measurements which can be obtained by using the diffraction precision index (DPI; Gurusaran *et al.*, 2014 ▸; Kumar *et al.*, 2015 ▸). The combination of using small-molecule rhenium crystallographic data from the CSD with protein crystallography allows us to pinpoint the coordination mode (*i.e.* monodentate, bidentate or tridentate coordination). Both the iodoplatin and rhenium studies utilize X-ray absorption at an X-ray-absorbing centre. The iodoplatin studies allowed two X-ray absorption edges to be harnessed, the I *K* and Pt *K* edges, each offering different penetrating powers for X-rays into a tumour. The rhenium study here again relies on X-ray absorption, now with a higher total occupancy owing to interaction with more amino-acid types than previously observed. One parameter used extensively during this study was the ‘quasi bite angle’ (QBA), defined here as the angle formed between the rhenium metal and cognate amino-acid residue atoms, which gives increased insight to the binding mode compared with small-molecule rhenium bite angles and related bond distances.

Current protein crystallography studies reporting rhenium coordination are rare and report a binding preference for the histidine imidazole side chain (Binkley *et al.*, 2011 ▸; Zobi & Spingler, 2012 ▸; Santoro *et al.*, 2012 ▸; Takematsu *et al.*, 2013 ▸). For radiopharmaceutical applications, it was noted that coordination could be achieved by first reacting the *fac*-[Re(CO)_3_]^+^ core, followed by ligand coordination to the metal after protein binding (Zobi & Spingler, 2012 ▸; Santoro *et al.*, 2012 ▸). This inverted [2+1] approach (Mundwiler *et al.*, 2004 ▸) is troubling from the aspect of site-specific drug interaction, as the open coordination sites of *fac*-[*M*(CO)_3_]^+^ may first lead to high retention in the kidneys, liver and blood pool (Schibli *et al.*, 2000 ▸). The results obtained here in this study, whereby rhenium coordination is also observed to aspartic acid, glutamic acid, arginine and leucine residues, arising from the technical innovation of using tuned X-ray wavelengths to improve the detectability of Re-atom binding, opens new alternatives for preferential binding other than to an imidazole moiety of histidine alone.

## 2. Experimental   

### 2.1. Crystallization   

Standard crystallization conditions for hen egg-white lysozyme (20 mg) consisting of pH 4.7, 10% NaCl, 0.04 *M* sodium acetate with *fac*-[Et_4_N]_2_[Re(CO)_3_(Br)_3_] at 0.03 *M* in 1.4 ml water in sitting-drop conditions, initially without DMSO but with silicone oil as cryoprotectant, led to crystals with no diffraction. With the inclusion of DMSO at 7.5%(*v*/*v*), cryoprotection of the crystals with silicone oil yielded consistent and good diffraction. The use of Paratone oil caused decomposition of the crystals upon contact with the Paratone oil in conditions either without or with DMSO; this was plainly visible under a visible-light microscope. Pure silicone oil was thus utilized. The crystal was transferred into the oil on a microscope slide and moved for ∼3 s to allow complete coating. The crystals grew over a period of approximately three weeks. DMSO is typically used as a ‘drug vehicle’ to increase the *in vitro* and *in vivo* solubility of pharmaceuticals within the bloodstream, to increase the accessibility of drugs to cell membranes and muscle tissue, and to allow them to cross the blood–brain barrier (typically at dosages below 6 ml kg^−1^; Kelava *et al.*, 2011 ▸; Swanson, 1985 ▸; Santos *et al.*, 2003 ▸; Colucci *et al.*, 2008 ▸). Therefore, our *in vitro* crystallization condition has a clinical context, although the use of DMSO in the clinical context still remains controversial (Hall *et al.*, 2014 ▸). Carbonyl ligands in the coordinated *fac*-[Re(CO)_3_]^+^ to HEWL were directly confirmed as measured by their signature stretching frequencies in solid-state infrared spectra (IR, cm^−1^): v_(CO)_ = 2011, 1997, 1863.

### 2.2. X-ray data collection, structure solution and refinement   

The rhenium–HEWL data collection was first conducted on an in-house Bruker PLATINUM^135^ detector with the crystal sample at a distance of 50 mm from the detector. An X-ray exposure time of 10 s per 0.5° crystal rotation angle was used with an X-ray wavelength of 1.5418 Å. The data were processed using the Bruker *PROTEUM*2 internal software package. X-ray data were also collected on beamline I04 at Diamond Light Source (DLS) using an X-ray wavelength of 0.9763 Å so as to optimize the rhenium *f*′′ anomalous signal at the Re *L*_I_ absorption edge. Both of the X-ray diffraction data collections were carried out at a fixed temperature of 100 K for the samples. Data and space-group validation were further confirmed with *Zanuda* and *MOSFLM* (Lebedev & Isupov, 2014 ▸; Leslie & Powell, 2007 ▸; Battye *et al.*, 2011 ▸) in the *CCP*4 software suite. The structures were solved *via* molecular replacement, using the reported lysozyme structure with PDB code 2w1y (Cianci *et al.*, 2008 ▸) as a molecular-replacement search model within *Phaser* (McCoy *et al.*, 2007 ▸), and were then refined in *REFMAC*5 (Vagin & Teplyakov, 2010 ▸) in *CCP*4*i* (Winn *et al.*, 2011 ▸). Protein model refinement was initially conducted in the tetragonal space group *P*4_3_2_1_2. Owing to apparent unresolvable symmetry indications near the Leu129 residue, space-group validation was considered in triclinic (*P*1) and then orthorhombic (*P*2_1_2_1_2_1_) symmetry utilizing *Zanuda* (Lebedev & Isupov, 2014 ▸) and *MOSFLM* (Leslie & Powell, 2007 ▸) in the *CCP*4 software suite (Winn *et al.*, 2011 ▸). The orthorhombic *P*2_1_2_1_2_1_ space group was selected based on reasonable rhenium–amino-acid bond distances as found in the CSD and better clarity of the electron density for the coordinated ligands. The *R*_int_ values were basically indistinguishable [0.077 (1.453) in *P*2_1_2_1_2_1_ *versus* 0.077 (2.066) in *P*4_3_2_1_2], which we attribute to the protein being in the higher symmetry, while the lower symmetry space group is a better choice for the rhenium-compound binding with respect to feasible bond distances. The successful placement of two individual protein subunits using the same search model (PDB entry 2w1y) was a direct confirmation of the crystal-packing layout. A similar orthorhombic space-group allocation has indeed been seen before, for example in an HEWL structure containing cisplatin (Tanley *et al.*, 2012 ▸). With respect to the archived data, the authors make the following statement of rationale for the finalized data files that we have firstly deposited in the PDB and secondly deposited as Supporting Information to this article, guided by a referee and the editor as well as our own views.

The PDB is focused on the protein. The protein refines best, with the most sensible ADPs, in the tetragonal space group. In the orthorhombic space group, the protein ADPs are flagged as poorer by *PARVATI* (http://skuld.bmsc.washington.edu/parvati/; Merritt, 1999 ▸), but the rhenium–ligand distances and the best defined electron density refined in the orthorhombic space group more sensibly fit the prior knowledge from the CSD. To resolve this dichotomy, and to respect everyone’s wish to deposit an acceptable set of coordinates in the PDB, whilst also not compromising the prior knowledge in the CSD, we deposited in the PDB the tetragonal refined coordinates for the synchrotron data with the Re atoms but with the ligand atoms removed. The ligand model of the rheniums bound to the protein is however best served in the orthorhombic space group, and we deposit as Supporting Information to this article the best model, both chemically and with respect to fit to the X-ray diffraction data, along with the synchrotron diffraction data. Since the home-laboratory Cu *K*α data provided an important second X-ray wavelength, and were therefore important in decisions about Re-atom placement, but were not concerned with protein details, we provide the orthorhombic refined home-laboratory data model and structure factors with this article as Supporting Information. Therefore, in summary, the best protein model refinement has been deposited in the PDB (synchrotron) with the highest symmetry for the protein (tetragonal). The best model for the rhenium ligand–protein inter­actions is attached to this article (synchrotron, ortho­rhombic).

Model building and adjustment were conducted within the *Coot* molecular-graphics program (Emsley & Cowtan, 2004 ▸) alternating with cycles of *REFMAC*5 in *CCP*4*i*. Alternatively, refinement for software comparison was conducted in *PHENIX* (Afonine *et al.*, 2012 ▸). Ligand-binding occupancies were calculated using *SHELXTL* (Sheldrick, 2008 ▸), with further manual adjustment guided by residual *F*_o_ − *F*_c_ electron-density peak evidence.

The ‘quasi bite angle’ (QBA), in addition to the specific bond distances measured (and supported by the diffraction precision index; Gurusaran *et al.*, 2014 ▸; Kumar *et al.*, 2015 ▸), provides increased insight into the binding mode when compared with the small-molecule rhenium bite angles and related bond distances. We have also made extensive use of the Cambridge Structural Database using the rigorous search tools that the CSD provides.

The PDB deposition code for the DLS tetragonal refinement is 5nbj. In each refinement the finalized protein model coordination and the respective diffraction data sets (structure factors) and the PDB validation reports were provided to the editor for their use and for the referees. The raw diffraction images for the synchrotron and home laboratory data sets are available at Zenodo (Brink & Helliwell, 2017). Table 1 ▸ provides a summary of the data and the model refinements: column 1, tetragonal, synchrotron data, model of the protein with rheniums (deposited in the PDB); column 2, ortho­rhombic, synchrotron data, model of the protein refined with rheniums and ligands; column 3, home-laboratory Cu *K*α data, model of the protein refined with rheniums and ligands.

Additional refinement protocols and tables discussing various aspects are included in the Supporting Information. Supplementary Table S1(*a*): comparison of the effects on *F*_o_ − *F*_c_ residual electron-density map peaks by the various refinement programs of *SHELXL*, *PHENIX* and *REFMAC* including the proper utilization of rhenium *f*′ and *f*′′ values. Supplementary Table S1(*b*): comparison of the effects on *F*_o_ − *F*_c_ residual electron-density map peaks by the various refinement programs of *PHENIX* and *REFMAC* involving orthorhombic and tetragonal refinements. Supplementary Table S2: comparison of anomalous difference electron-density peak heights in the Cu *K*α and DLS (λ = 0.9763 Å) diffraction data. Supplementary Table S3: table of Re-atom distances from their cognate specific residues as well as their metal occupancies and *B* factors for Cu *K*α diffraction data. Supplementary Table S4: table of selected bond distances and angles derived from the Diamond Light Source (λ = 0.9763 Å) diffraction data set for the cases not discussed in the main text.

## 3. Results and discussion   

The coordination complex *fac*-[Re(CO)_3_(H_2_O)_3_]^+^ readily coordinates to monodentate, bidentate and tridentate ligand systems *via* the three labile aqua sites (Alberto *et al.*, 1999 ▸; Salignac *et al.*, 2003 ▸; Grundler *et al.*, 2004 ▸, 2006 ▸; Helm, 2008 ▸; Brink *et al.*, 2014 ▸). The facially coordinated carbonyl ligands have chemically known coordinative stability and it is conventionally considered to be chemically unlikely that any substitution will occur at these three sites unless specific carbonyl substitution is targeted (Tisato *et al.*, 2006 ▸; Braband *et al.*, 2012 ▸; Rattat *et al.*, 2001 ▸). Owing to chemical kinetic understanding of the stability of the rhenium tricarbonyl complexes, we have assumed that no exchange or substitution is currently occurring under our experimental conditions and therefore the occupancies of the ligands are the same as those of the Re atoms. Henceforth, the ligands where 2*F*_o_ − *F*_c_ electron density is present (*i.e.* His15) were refined with the same occupancy value as the Re atom in the orthorhombic refinement. The occupancy of any ligands in a cif monomer which show partial but not complete 2*F*_o_ − *F*_c_ electron density have been refined with an occupancy of zero. This was performed in order to abide with the current chemical and kinetic understanding of *fac*-[Re(CO)_3_(H_2_O)_3_]^+^ complexes whilst respecting the weak electron density (presumed to be due to ligand mobility). In sites where the position of the rhenium metal centre is clearly defined by the anomalous difference density map, *i.e.* Leu129, but where no/little 2*F*_o_ − *F*_c_ electron density occurs above the 1.3σ level, only the position of the rhenium metal has been placed.

**Figure 1:**
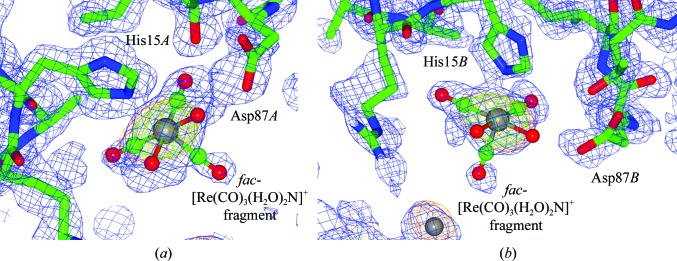
Composite OMIT maps of the binding site at His15 in chains *A* (*a*) and *B* (*b*) coordinating to *fac*-[Re(CO)_3_(H_2_O)_2_N] (the N atom is from His15). The 2*F*_o_ − *F*_c_ electron-density map contoured at 1.2 r.m.s. is shown in blue and the *F*_o_ − *F*_c_ electron-density map contoured at 5.0σ (the *Coot* default; Emsley & Cowtan, 2004 ▸) is shown in green; the anomalous electron-density map contoured at 3.0σ is shown in orange. This figure was prepared using *CCP*4*mg* (McNicholas *et al.*, 2011 ▸).

Rhenium binding to aspartic acid occurs at residues Asp119*A*, Asp18*A*, Asp18*B*, Asp52*A* and Asp52*B*. Binding to glutamic acid occurs at residues Glu7*B*, Glu35*A* and Glu35*B*. Binding to arginine occurs at residues Arg125*B* and Arg61*B* (however, the position of Arg61*B* is relatively uncertain owing to poor 2*F*_o_ − *F*_c_ density). Binding to leucine occurs at the Leu125*A* and Leu129*B* residues. Rhenium complexes also occur in the vicinity of residues Leu129*B*, between Pro70*A* and Arg61*A*, and in the region of Arg14*B* as nonbinding entities. We note therefore the large number of repeated binding observations at identical amino-acid types in the protein *A* and *B* subunits. We confirm the octahedral environment of the rhenium at the His15 residues in both the *A* and *B* subunits of the protein (Fig. 1 ▸), which has the highest refined metal occupancy. Where the metal occupancy is lower, not all of the ligand positions of *fac*-[Re(CO)_3_(H_2_O)_*n*_]^+^ (*n* ≤ 3) are defined by electron density. We have therefore refined with a monomer cif file (labelled as RRE) in sites which show incomplete electron density for the coordinative ligands, or have simply placed the Re atom in position according to the anomalous density map, as its core evidence.

The rhenium tricarbonyl complex coordinates to the His15*A* and His15*B* side chains, with bond distances of 2.25 (8) and 2.36 (8) Å. The electron-density map is well defined around His15*A*, whereas a break in the 2*F*_o_ − *F*_c_ density along the *trans* axial carbonyl ligand is observed for His15*B*. Related small-molecule *fac*-[Re(CO)_3_N_imidazole_] complexes show bond distances ranging from 2.174 (4) to 2.197 (5) Å (Schibli *et al.*, 2000 ▸; Garcia *et al.*, 2000 ▸; Fernández-Moreira *et al.*, 2014 ▸; Brink *et al.*, 2013 ▸). The protein study here and the CSD values therefore agree within statistical precision, which is naturally limited by the 1.26 Å resolution X-ray model refinement of the protein. The occupancies of the Re atoms are 83% for both chains *A* and *B* (Fig. 1 ▸). Reports by Binkley *et al.* (2011 ▸) and Zobi & Spingler (2012 ▸) similarly mention preferential binding to the histidine imidazole moiety and describe well the structural environment around this rhenium tri­carbonyl fragment as well as the ligand coordination.

**Figure 2:**
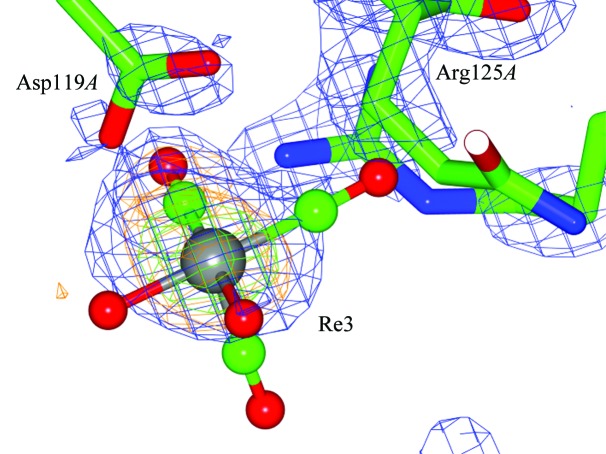
Composite OMIT maps of the Asp119*A* binding site coordinating to *fac*-[Re(CO)_3_(H_2_O)_2_]. This figure clearly shows the close proximity of the Arg125 residue to the rhenium core. The electron-density maps are contoured as in Fig. 1 ▸. *F*_o_ − *F*_c_ density is indicated for the axial CO and H_2_O ligands, which are refined according to the monomer cif library. However, no electron density is apparent for the *trans* coordinating CO ligand. The CO has therefore been refined with zero occupancy but must be chemically present, in accordance with the current chemical understanding of *fac*-[*M*(CO)_3_]^+^ complexes as previously stated. The quasi bite angle (QBA) at Asp119*A* is 41 (2)° for OD2—Re3H⋯OD1. The distances to the Re atom are 2.5 (1) and 3.26 (9) Å, respectively. The coordination environment at Asp119*B* is similar, with bond distances as listed in Supplementary Table S4. This figure was prepared using *CCP*4*mg* (McNicholas *et al.*, 2011 ▸).

Rhenium binding to aspartic acid residues occurs repeatedly, namely Asp119*A* (Fig. 2 ▸), Asp18*A* and Asp18*B*, and Asp52*A* and Asp52*B*. Close proximity of Arg125*A* NH_2_ to the O atom of the carbonyl ligand [2.13 (8) Å] is observed for the Re3H complex (also visible in Fig. 2 ▸). The interspatial distance [3.96 (9) Å] between the respective NH_2_ and Re atom is, within the bond-distance error of 0.09 Å, a van der Waals interaction (*i.e.* 3.7 Å). Similarly, for chain *B* a close inter­action is observed between Arg125*B* NH_2_ and the Re atom [NH_2_⋯Re4D = 1.98 (9) Å], which likewise in turn shows coordination to Asp119*B*.

An unusual cyclic dimer is found in the CSD (CSD refcode UDENAU) in which one Re atom binds bidentately to the N (α-amino) and O (α-carboxylate) atoms of an aspartic acid subunit (Supplementary Fig. S1). The third available position is occupied by the O atom (β-carboxylate) from a second Asp unit (Nayak *et al.*, 2013 ▸). The Re—O_1_(β-carboxylate) and Re-O(α-carboxylate) bond distances are both 2.149 (4) Å. The distance between the noncoordinated O_2_(β-carboxylate) and the Re atom is 3.35 Å. The QBA for O_1_(β-carboxylate)—Re⋯O_2_(β-carboxylate_noncoordinated_) is measured as 41.5°, a very close approximation to the QBA between Re and Asp119. A short contact of 3.306 (8) Å is observed for the dimer between the (α-amino) NH_2_ and the CO ligand of the neighbouring rhenium complex. Another small-molecule crystal structure in the CSD is an aspartic *N*-monoacetic acid coordinated tridentately to *fac*-[Re(CO)_3_(H_2_O)_3_]^+^, which has average Re—O bond distances of 2.138 (2) Å (CSD refcode CEJSOB; Klenc *et al.*, 2012 ▸); these are within the range of that found in this protein crystallographic study.

**Figure 3:**
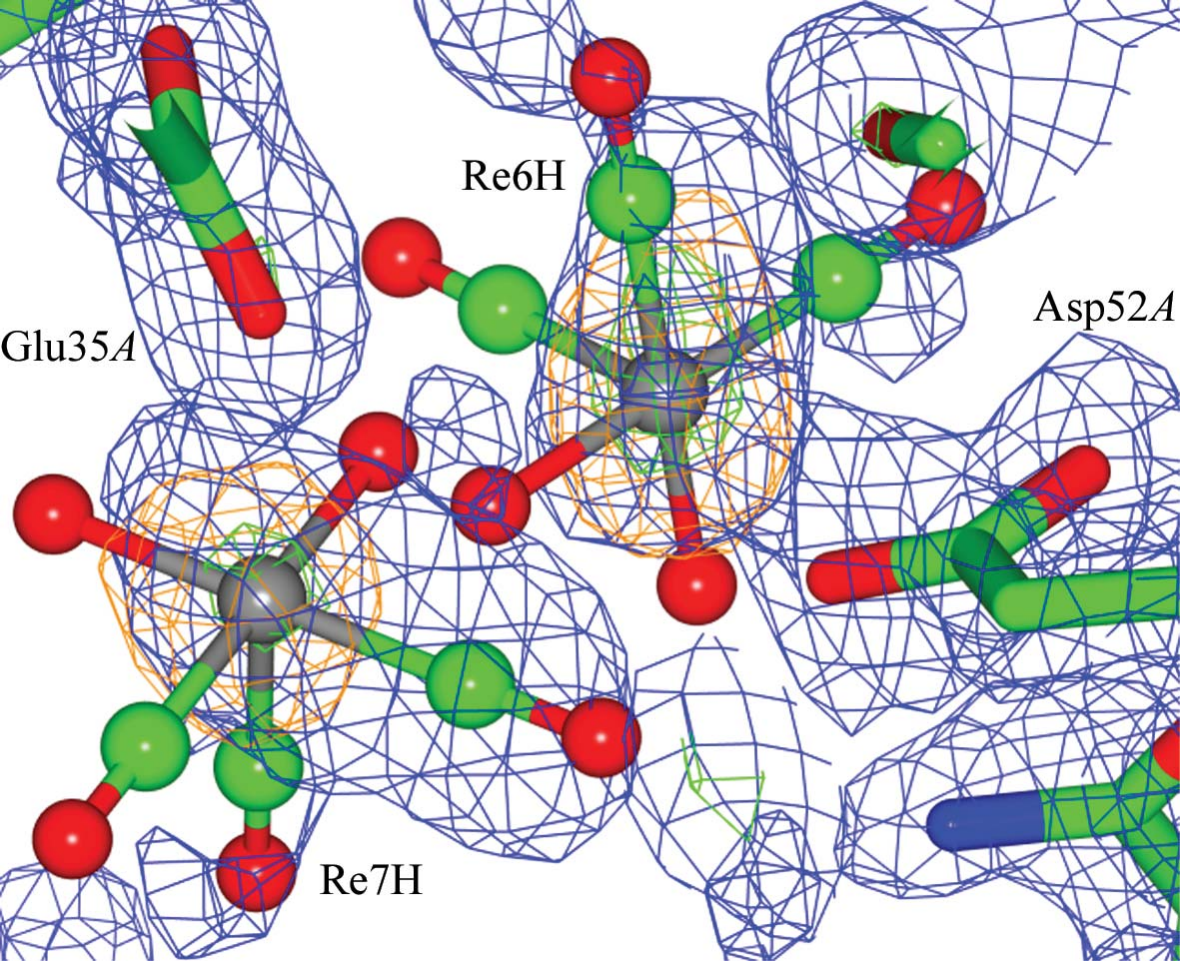
Composite OMIT maps of the Asp52*A* and Glu35*A* binding sites coordinating to *fac*-[Re(CO)_3_(H_2_O)_2_]^+^, indicating the close proximity of the two Re atoms to each other. The relative positions of the Re atoms are clearly defined by the anomalous map to σ levels of 14.3 and 14.8. Clashes occur between the carbonyl and aqua ligands of the two Re atoms. The respective orientation of the *fac*-[Re(CO)_3_]^+^ moieties has been placed as well as possible, taking into account the limited 2*F*_o_ − *F*_c_ density. An *F*_o_ − *F*_c_ electron-density peak (6σ) is found in the vicinity, but its assignment is chemically uncertain and was therefore not made. The likely explanation for this layout, since their summed occupancies is less than 100%, is that a fraction of the unit cells in the crystals have a rhenium in one location and another fraction favours the nearby location. An alternative is that it is a dirhenium compound, for which there are several possibilities such as oxo-bridged, carbonyl bridged or metal–metal complexes. Electron-density maps are contoured as in Fig. 1 ▸. This figure was prepared using *CCP*4*mg* (McNicholas *et al.*, 2011 ▸).

The rhenium metal atom coordination at Asp18*A* has an occupancy of 33%. The bond distances to the Re atom are 2.06 (9) and 3.00 (8) Å, with a QBA of 47 (2)°. The coordination at Asp52*A* has an occupancy of 42%. The bond distances from the carboxylic O atom to the Re atom are 2.2 (1) and 3.5 (1) Å, with a QBA of 34 (1)°. A second rhenium complex coordinated to Glu35*A* (rhenium occupancy 30%) lies in close proximity, with definable *F*_o_ − *F*_c_ density and an Re—OE1 bond distance of 2.62 (8) Å, a distance which is shorter than the sum of the van der Waals radii (3.67 Å; Desiraju & Steiner, 2006 ▸). Interestingly, the rhenium metal centres of Re6H and Re7H are separated by 3.8 (1) Å, which is also within the range of a van der Waals interaction (4.3 Å; Figs. 3 ▸ and 4 ▸).

Related small-molecule structures of *fac*-[Re(CO)_3_]^+^ bound to aspartic acid fragments have Re—O bond distances varying from 2.138 (2) to 2.149 (4) Å (Klenc *et al.*, 2012 ▸; Nayak *et al.*, 2013 ▸), whereas the Re—OH_2_ bond distances for small-molecule *fac*-[Re(CO)_3_(H_2_O)_3_]^+^ complexes typically vary from 2.171 (5) to 2.21 (1) Å (Alberto *et al.*, 1999 ▸). Bite angles that are formed from four coordinated κ^2^O,O′ complexes range from 59.4 (1) to 59.8 (2)° (Gibson *et al.*, 1994 ▸, 1999 ▸).

An Re atom is found in the vicinity of Glu7*B* with a distance of 3.30 (8) Å between the OE2 and Re atoms. Direct coordination is considered to be unlikely owing to the reported Re—O bond distance of the small-molecule Re(κ-N,O) glutamic acid complex (Mundwiler *et al.*, 2004 ▸) being 2.163 (4) Å (CSD refcode EXIWUD). However, a possible interaction may occur between the atoms as the distance is less than the sum of the van der Waals radii (3.67 Å). Only the placement of the Re atom is defined, as no 2*F*_o_ − *F*_c_ density is observed for the aqua or carbonyl ligands (the anomalous difference peak height from the DLS data is 8.3σ *versus* 4.7σ for the Cu *K*α data), *i.e.* as expected owing to the X-ray wavelength optimization of the rhenium *f*′′, thus further confirming its identification.

Additionally, binding to arginine is observed at Arg61*B*, with respective Re—N distances of 2.4 (2) Å and (a not chemically possible) 0.8 (2) Å (NH_1_); however, the positions of Arg61*B* and Arg61*A* are relatively uncertain owing to poorly defined 2*F*_o_ − *F*_c_ density, in particular for NH_1_. The occupancy of the Re atom is 40% [anomalous difference peak-height evidence from DLS data = 9.4σ (Arg61*A*) and 9.7σ (Arg61*B*)]. There are, however, to date no small-molecule crystal structures of *fac*-[M(CO)_3_]^+^ (*M* = Re, Tc) bonded to arginine listed in the CSD to allow a direct comparison (Allen, 2002 ▸).

Binding to leucine occurs at the Leu129*A* and Leu129*B* residues, with Re—O bond distances of 2.5 (1) Å (occupancy of 31%) and 2.8 (1) Å (occupancy of 21%), respectively. A van der Waals interaction occurs between the Leu129*B* OXT atom and Re [spatial distance of 3.23 (8) Å, which is less than the sum of the van der Waals radii (3.67 Å)]. No rhenium(I)–leucine complexes are found in the CSD database; however, a rhenium(V)–leucine complex (Melián *et al.*, 2000 ▸) (CSD refcode LOPTOZ) has been reported with typical Re—O_leucine_ and Re—N_leucine_ bond distances of 2.12 (1) and 2.22 (3) Å, respectively.

**Figure 5:**
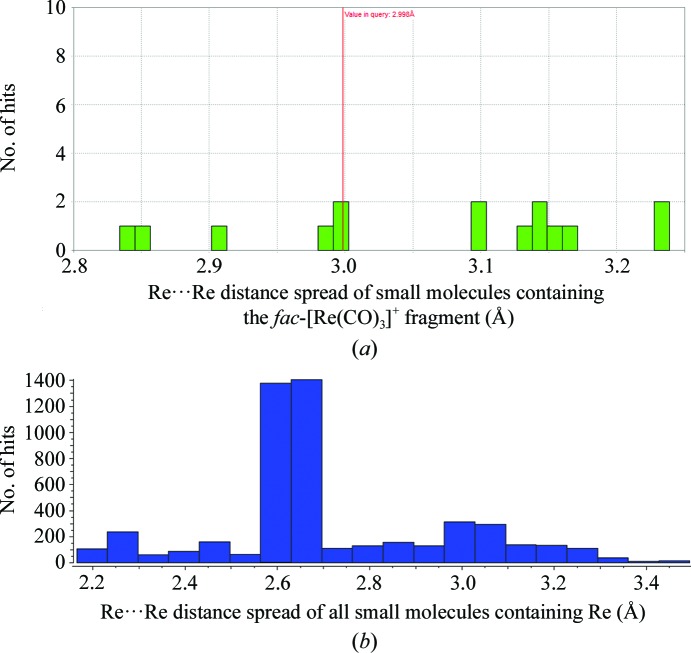
Comparison of all possible small-molecule Re⋯Re interactions. (*a*) *Mogul* search plots of the number of small-molecule hits utilizing Re⋯Re interactions containing the *fac*-[Re(CO)_3_]^+^ fragment as a search criterion found in the CSD database. Colour bars indicate the number of structure hit entries with respect to bond length (Å) in the CSD version update 5.37 data library. The minimum/maximum bond distances had a standard deviation of 0.129 Å and a mean value of 3.065 Å. (*b*) Data analysis of all small-molecule hits {not necessarily containing the *fac*-[Re(CO)_3_]^+^ fragment as illustrated in (*a*)} for Re⋯Re interactions as a search criterion in the CSD database. The longest Re⋯Re distance [3.4934 (6) Å] is found in a cyclic pentakis­(μ-hydrido)-icosacarbonyl-pentarhenium complex (CSD refcode PORYIE; Bergamo *et al.*, 1998 ▸), whereas the shortest is for a carboxylate-dirhenium complex (CSD refcode GUVTUO; Golichenko & Shtemenko, 2015 ▸).

Rhenium anomalous difference density also occurs in the vicinity of residues Leu129*B* [3.47 (7) Å], where a 40% occupancy rhenium complex occurs. The electron density is partially explained by the allocation of two fractionally disordered molecules, the positions of which have an Re⋯Re distance of 1.5 (1) Å as indicated by the anomalous map. The complex is unlikely to be a Re—Re chemical dimer owing to the too short distance value and the stable oxidation state of *fac*-[Re^I^(CO)_3_]^+^ as well as the following results from the CSD. Utilizing *Mogul* (Bruno *et al.*, 2004 ▸) to search for all possible Re⋯Re interactions involving the *fac*-[Re(CO)_3_]^+^ fragment yields 15 hits in the CSD with minimum/maximum bond distances ranging from 2.837 to 3.233 Å (Fig. 5 ▸ *a*). If all Re⋯Re interactions are considered, irrespective of their oxidation states and coordination mode, a search yield of 1546 hits is obtained with minimum/maximum bond distances ranging from 2.245 to 3.497 Å (Fig. 5 ▸ *b*).

In addition to the diversity of binding to these various amino acids, as described in detail above, we also note that nonbonding rhenium entities are found between residues in the vicinity of Pro70*A* [Re⋯O = 3.6 (1) Å] and Arg61*A* with an occupancy of 60% and in the region of Arg14*B* [Re⋯N = 6.2 (1) Å] with an occupancy of 40%.

## 4. Conclusions   

In summary, we have found multiple binding sites for a rhenium metal complex on different amino acids, findings that have never been reported previously. Specifically, we see that there are numerous chemical coordination possibilities for the fundamental synthon *fac*-[Re^I^(CO)_3_(H_2_O)_3_]^+^ to Asp, Glu, Arg and His amino-acid residues as well as to the C-terminal carboxylate in the vicinity of Leu and Pro. Naturally, we confirm that the highest occupancy binding is to the histidine imidazole group. Rhenium and technetium have similar chemical properties but distinctly different radioactive signatures, with ^188^Re and ^186^Re being used for therapy and ^99m^Tc being used for imaging across all diseases. This diversity of amino-acid interactions that we have uncovered, in addition to the ability to precisely indicate bond-distance and angle ranges for specific three-dimensional structure development, will create more new Re/Tc lead compounds for site-specific binding to protein tissue in radiopharmaceutical applications. Furthermore, the total sum of the binding of rhenium to the various amino acids that has been discerned would immediately lead to the possibility of a reduced, or better yet targeted, radiation dose with respect to medical radiation treatment.

## Supplementary Material

PDB reference: hen egg-white lysozyme with bound rhenium, 5nbj

Supplementary Information. DOI: 10.1107/S2052252517003475/lt5001sup1.pdf

Diamond Light Source orthorhombic data. Zip file contains DLS orthorhombic protein crystallographic structure factors, coordinates, ligand cif file and anomalous density map.. DOI: 10.1107/S2052252517003475/lt5001sup2.zip

Cu K alpha orthorhombic data. Zip file contains structure factors, coordinates, ligand cif file and anomalous map.. DOI: 10.1107/S2052252517003475/lt5001sup3.zip

DLS tetragonal refinement : data submitted to PDB (PDB entry 5nbj).. DOI: 10.1107/S2052252517003475/lt5001sup4.zip

Raw diffraction images URL: https://doi.org/10.5281/zenodo.345364

## Figures and Tables

**Figure 4:**
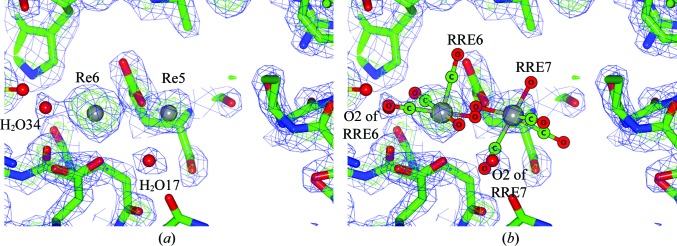
Anomalous density accuracy in an overlay fit between the Cu *K*α and DLS models. (*a*) The composite OMIT electron-density map for the Cu *K*α laboratory data at Re6 and Re5, illustrating the relative positions of the Re atoms (as determined by the anomalous difference density map) and water solvent molecules (H_2_O34 and H_2_O17, as determined by the *F*_o_ − *F*_c_ and 2*F*_o_ − *F*_c_ electron-density maps). (*b*) Identical view as in (*a*) for the DLS data. An overlay fit between the Cu *K*α and DLS models gave an r.m.s.d. of 0.14 Å for this subunit, *i.e.* closely identical in all respects. The *fac*-[Re(CO)_3_(H_2_O)_2_(X)] cif file (RRE) indicating the positions of the Re atoms in the DLS data as determined by the DLS X-ray wavelength optimized anomalous difference electron-density map. Notice the near-perfect overlay position of the respective Re atoms, *i.e.* Re6 and Re5 *versus* RRE6 and RRE7, as well as for H_2_O34 and H_2_O17 *versus* O2 for RRE6 and RRE7.

**Table 1:** X-ray crystallographic data and final protein model refinement statistics for Diamond Light Source (DLS) data (refined in tetragonal and orthorhombic space groups) and Cu *K*α data (orthorhombic) Overall diffraction resolution values are given, with values for the outer diffraction resolution shell in parentheses.

	DLS (λ = 0.9763 Å) (tetragonal; PDB code 5nbj)	DLS (λ = 0.9763 Å) (orthorhombic)†	Cu *K*α (λ = 1.5418 Å) (orthorhombic)†
Data reduction			
Space group	*P*4_3_2_1_2	*P*2_1_2_1_2_1_	*P*2_1_2_1_2_1_
Unit-cell parameters‡ (Å, °)	*a* = 79.89 (1), *b* = 79.89 (1), *c* = 37.00 (2), α = β = γ = 90	*a* = 36.98 (3), *b* = 79.80 (1), *c* = 79.92 (1), α = β = γ = 90	*a* = 79.70 (1), *b* = 79.71 (1), *c* = 36.83 (3), α = β = γ = 90
Molecular mass (Da)	14700	14700	14700
Molecules per asymmetric unit	1	2	2
Detector	Dectris PILATUS 6M-F	Dectris PILATUS 6M-F	Bruker APEX II
Crystal-to-detector distance (mm)	135	135	40
X-ray wavelength (Å)	0.97625	0.97625	1.5418
Observed reflections	735148 (31186)	735464 (99591)	647723 (22460)
Unique reflections	32463 (1660)	63838 (9126)	22610 (3107)
Resolution (Å)	39.95–1.27	56.47–1.26	39.86–1.79
Completeness (%)	99.9 (98.3)	99.9 (99.5)	99.4 (96.4)
*R*_merge_	0.077 (2.066)	0.077 (1.453)	0.142 (0.750)
〈*I*/σ(*I*)〉	20.9 (1.7)	14.7 (1.6)	17.52 (1.92)
Multiplicity	22.6 (18.8)	11.5 (10.9)	28.48 (10.9)
Mn(*I*) half-set correlation CC_1/2_	0.999 (0.556)	0.998 (0.536)	§
Cruickshank DPI (Å)	0.049	0.050	¶
Average *B* factor (Å^2^)	21.0	22.8	20.45
Refinement			
*R* factor/*R*_free_ (%)	17.22/19.6	17.9/22.6	19.4/26.6
*R* factor, all (%)	17.22	18.2	16.6
R.m.s.d., angles (°)	1.145	2.793	1.122
Ramachandran values (%)			
Most favoured	98.4	96.6	98.8
Additional allowed	1.56	3.44	1.16
Disallowed	0	0	0

† The raw diffraction images are available at Zenodo (Brink & Helliwell, 2017 ▸).

‡ Note that the order of the *a*, *b*, *c* unit-cell parameter values in Table 1 ▸ follows the respective conventions of the two different diffraction data-processing programs that we have used.

§ The CC_1/2_ metric is more recently introduced than the Bruker software used with the APEX II instrument, which therefore does not include it. The other, much used, metric of 〈*I*/σ(*I*)〉 crossing 2 is provided.

¶ In the case of anisotropic protein model refinement undertaken at a diffraction resolution worse than ∼1.6 Å the calculated DPI formula denominator value of [number of observations (21313) − number of refined parameters (20690)] is approaching zero and the DPI estimate thus becomes unstable. Therefore, the distance values from our Cu *K*α data cannot have reliably reported e.s.d. values. Details regarding the ‘DPI webserver’ can be found in Kumar *et al.* (2015 ▸). We prefer to use an anisotropic refinement for the Cu *K*α case as it improved the *F*_o_ − *F*_o_ residual density, in particular around the Re atoms and their coordinated ligands.
