# Supplementary material for: New leads for fragment-based design of rhenium/technetium radiopharmaceutical agents
Source: IUCrJ. 2017 Apr 11;4(Pt 3):283–90. doi: 10.1107/S2052252517003475 (PMC5414402; doi:10.1107/S2052252517003475)
Supplement: Supplementary file 1 [file m-04-00283-sup1.pdf]

IUCrJ

Volume 4 (2017)

Supporting information for article:

New leads for fragment-based design of rhenium/technetium radiopharmaceutical agents

Alice Brink and John R. Helliwell

Table S1

(a) Comparison of Fo-Fc residual electron density map peaks for *fac*-[Re(CO)$_3$]$^+$ metal occupancy (Occ.) refinement based on X-ray diffraction data recorded on beamline I04 at the Diamond Light Source (DLS) (λ = 0.9763 Å) in the orthorhombic spacegroup. Whilst the Shelxl refinement of occupancies and B factors, and including the rhenium f ‘ and f “ values (second group of columns), is preferred we noted the residual Fo-Fc peaks at the rhenium sites. We therefore undertook a manual adjustment of occupancy values and subsequent Refmac refinement of their B values and which improved these i.e. led to smaller residual peaks (fourth group of columns). The third group of columns shows the Phenix refine estimates. There is a close agreement across these three methods. The first group of columns are purely to illustrate that (incorrect use of f ‘ and f “ values of zero) do have an effect and the proper f ‘ and f “ values must be allowed for.

**Rhenium's with Subunit B** [g]	Shelxl refinement [a]			Shelxl refinement factoring in f' and f'' for λ 0.9763 Å [b]			Phenix refinement [c]			Manually adjusted then Refmac refinement [d]		
	Occ.	Multiples of σ (Fo-Fc)	B factor	Occ.	Multiples of σ (Fo-Fc)	B factor	Occ.	Multiples of σ (Fo-Fc)	B factor	Occ.	Multiples of σ (Fo-Fc)	B factor
RRE 1	75.3(2)	6.3	30	71.9(1)	7.2	30	81	5.4	32	83	5.0	31
RRE 2	72.4(1)	6.8	29	69.2(1)	8.5	29	81	5.3	32	83	4.4	30
RRE 3	36.4(8)	6.3	29	34.91(5)	6.2	30	32	4.2	30	38	5.8	31
RRE 4	33.0(1)	4.6	29	21.5(1)	5.4	26	38	5.1	32	33	4.2	28
RRE 5	31.4(1)	4.5	29	29.75(4)	4.8	29	35	5.0	33	32	5.0	30
RRE 6	31.3(1)	14.2	82	28.4(1)	12.6	75	42	10.0	78	42	10	128
RRE 7	28.9(1)	6.0	42	27.5(1)	4.6	41	36	6.5	58	30	6.0	39
RRE 8	23.7(2)	5.4	25	22.7(2)	5.7	27	33	-11.0	38	23	4.8	24
RRE 9	27.0(1)	15.9	73	25.1(2)	12.8	62	44	-8.7	49	42	8.3	179
RRE10A	40.3(2)	3.0	19	40.5(2)	3.0	19	33	6.0	20	40	3.0	19
RRE10B	37.2(1)	-5.5	38	41.2(1)	-6.3	40	39	-6.6	47	37	4.0	37

**Rhenium's with Subunit D** [g]												
Re 4	34.6(1)	4.8	31	34.86(4)	5.0	32	32	4.3	33	35	5.0	32
Re 7	31.0(1)	3.0	39	30.6(1)	2.7	39	31	3.5	42	31	3.0	38
Re 8	20.7(1)	3.0	37	22.2(2)	3.9	37	26	4.3	43	21	4.0	36
Re 13	31.(1)	12.0	88	31.93(3)	20.0	122	59	6.5	95	60	17	146
Re 14	29.6(1)	4.8	117	30.59(4)	6.9	162 [f]	39	4.1	106	40	4.0	243 [e]
Re 15	17.7(1)	4	24	17.19(6)	3.8	25	18	3.7	27	18	4.0	25
Re 16	50.4(1)	-5	69	48.4(2)	-5.6	68	40	3.3	82	40	4.1	60

[a] R/Rf = 17.6/22.0; [b] R/Rf = 17.7/22.3; [c] R/Rf = 20.6/24.9 (Phenix refinement); [d] R/Rf = 17.2/22.0.

[e / f] These are estimates of the B-factor of Re 14 and by their nature, being large, are imprecise as is any case evident from the widely different values (162 *vs*. 243) from the two different programs with the same diffraction data. A similar situation occurs with RRE 9 and Re 13, albeit not as extreme. We believe this is testimony to both programs (Phenix and Refmac) giving basically the same physically reasonable values to the same diffraction data, and we think that consistency between the two is good.

[g] Rhenium atom refinements defined in subunits B and D as labelled in the PDB file.

Notes:

1. The naming of the rhenium atoms is according to peak height ranking number in the DLS data anomalous difference map peak height list and therefore obviously does not exactly match the Cu Kα anomalous peak height ranking list. The closest residue is therefore listed as the reference marker for the reader.
2. Table footnote [d]: "Manual" means the Re metal atom occupancies were manually adjusted until there was a minimised Fo-Fc difference electron density at each of these sites. Our efforts to fully flatten to zero the Fo-Fc in the manual adjustment Refmac refinement final step were largely successful (residual Fo-Fc peaks on the rhenium atoms being between 3 and 5 σ, which are typically below a water molecule at around 6 σ, one water molecule obviously being a total of 10 electrons). The exceptions were the few rhenium's with high B factors which were sensitive to any adjustment.

(b) Additional comparison of Fo-Fc residual electron density map peaks for *fac*-$[Re(CO)_3]^+$ metal occupancy (Occ.) refinement based on X-ray diffraction data recorded on beamline I04 at the Diamond Light Source (DLS) (λ = 0.9763 Å). Columns compare the **Orthorhombic** refinement (**$P2_12_12_1$**; Phenix and Refmac Manual Refinement) versus the **Tetragonal** refinement (**$P4_32_12$**; Phenix) whereby only the rhenium atoms (no ligands) have been defined.

**Rhenium's with Subunit B**	Closest residue	Phenix refinement (Orthorhombic) [a]			Manually adjusted then Refmac refinement (Orthorhombic) [b]			Closest residue	Phenix refinement (Tetragonal)[c]		
		Occ.	Multiples of σ (Fo-Fc)	B factor	Occ.	Multiples of σ (Fo-Fc)	B factor		Occ.	Multiples of σ (Fo-Fc) [d]	B factor
RRE 1	His15A	81	5.4	32	83	5.0	31	His15A (Re1B) [1]	100	10.5	34
RRE 2	His 15B	81	5.3	32	83	4.4	30	[1]	-	-	-
RRE 3	Asp119A (..Arg125)	32	4.2	30	38	5.8	31	Asp119A (Re1D) [3]	36	7.3	30
RRE 4	Asp18A	38	5.1	32	33	4.2	28	Asp18A (Re1F) [4]	34	6.9	27
RRE 5	Asp18B	35	5.0	33	32	5.0	30	[4]	-	-	-
RRE 6	Asp52A	42	10.0	78	42	10	128	Asp52A (Re1K) [6]	23	6.5	26
RRE 7	Glu35A	36	6.5	58	30	6.0	39	Glu35A (Re1I) [7]	20	7.3	19
RRE 8	Glu35B	33	-11.0	38	23	4.8	24	[7]	-	-	-
RRE 9	Asp52B	44	-8.7	49	42	8.3	179	[6]	-	-	-
RRE10A	Leu129B	33	6.0	20	40	3.0	19	Leu129A (Oxt) (Re2B) [2]	50	-8.0	27
RRE10B	Leu129B	39	-6.6	47	37	4.0	37	Leu129A (Oxt) (Re3B) [2]	12	-8.0	64
**Rhenium's with Subunit D** [g]											
Re 4	Asp119B (…Arg125B)	32	4.3	33	35	5.0	32	[3]	-	-	-
Re 7	Leu129A	31	3.5	42	31	3.0	38	Leu129A (Re1H) [5]	32	6.1	39
Re 8	Leu129B	26	4.3	43	21	4.0	36	[5]	-	-	-
Re 13	Pro 70A	59	6.5	95	60	17	146	Pro70A (Re1M) [8]	40	5.6	52
Re 14	Pro70B	39	4.1	106	40	4.0	243 [e]	[8]	-	-	-
Re 15	Glu7B	18	3.7	27	18	4.0	25	Glu7A (Re1N)[9]	22	3.8	28

Re 16	Glu7A	40	3.3	82	40	4.1	60	[9]	-	-	-

[a] R/Rf = 20.6/24.9 (Phenix refinement, Orthorhombic); [b] R/Rf = 17.2/22.0 (Orthorhombic); [c] R/Rf = 16.9/19.4 (Phenix refinement, Tetragonal)

Note A. [d] As rhenium-ligand positions cannot always be definable due to lack of Fo-Fc density at the current resolution, no ligands have been refined in the tetragonal refinement, even in the case of His15 where the carbonyl and aqua density can be clearly observed. Only the positions of the rhenium atoms (by anomalous difference map peak heights) are defined. Additional Fo-Fc density can therefore be expected.

Note B. Numerical superscripts **1-9** indicate where the counterpart rhenium atoms could be found in the respective orthorhombic refinement versus the tetragonal refinement.

Note C. Initially, we of course analysed the protein and bound rhenium model in tetragonal. The highest occupied rhenium was bound to His15, as expected. But unexpected was the obviously incorrect Re to imidazole nitrogen His15 refined ligand distance in Refmac of 1.89 Å. The Refmac refinement made then in the lower, orthorhombic, symmetry yielded Re to His ligand distances in a good match to CSD values (2.17-2.19 Å). Another feature leading to preferring to continue our analyses in orthorhombic was the better clarity of the electron density for the ligands to the rhenium's. Finally, we also note a variation in both aspects, the Re to His15 refined ligand distance and ligand density peaks, between Refmac (1.86(8) Å) and Phenix (2.19(8) Å) refined tetragonal models. Overall, for the determination of the rhenium ligand binding details, which is our focus, our descriptions are primarily made in orthorhombic.

Table S2 The increase in anomalous differences electron density peak heights in the Cu Kα and DLS diffraction data due to the optimised diffraction wavelength.

**Residue**	**Cu Kα (λ = 1.5418 Å)**	**DLS (λ = 0.9763 Å)**
	Re *f* " = 5.9	Re *f* " = 12.1
His 15A	19.3	43.1
His 15B	19.5	43.8
Asp 18A	10.0	13.7
Asp 18 B	8.8	13.4
Asp 52A	6.9	12.9
Asp 52B	7.1	12.5
Asp 119A & Arg 125A	7.4	16.1
Asp 119B & Arg 125B	8.5	16.3
Leu 129A	3.2	9.0
Leu 129B	3.3	8.5
Glu 35A	4.8	12.2
Glu 35B	5.3	10.5
Near Pro 70A	4.6	9.4 (Arg 61A)
Near Pro 70B	4.8	9.7 (Arg 61B)
In vicinity of Leu 129B	10.5	A: 23.3 & B: 24.0
In vicinity of Glu 7B, Arg 14A & His 15A	4.7 (3.0 Å from Glu 7B)	8.3 (3.3 Å from Glu 7B)
In vicinity of Glu 7A, Arg 14B & His 15B	6.3 (3.4 Å from Glu 7A)	9.2 (3.5 Å from Glu 7A)

Note: the rhenium anomalous dispersion signal is f " of 12.1 electrons, at its L1 absorption edge with a selected X-ray wavelength of 0.9763Å. At Cu Kα X-ray wavelength (1.5418 Å) the Re f " is 5.9 electrons.

Table S3 Table of rhenium atom distances from their cognate specific residues as well as their metal occupancies and B factors for CuKα diffraction data.

Chain	Residue	Re label from file	Distance of residue from Re atom (Å) [a]	Manual Refinement (orthorhombic)	
				Occ. (%)	B factor (Å^2)
D1	His 15A	Re 1F	His 15 A – 2.4	100	33.1
D9	His 15B	Re 2F	His 15 B – 2.4	100	33.8
D3	Asp 18A	Re 3D	Asp 18A (O2) – 2.1<br>Asp 18A (O1) – 3.1<br>O-Re-O bite angle = 44°	50	28.0
D11	Asp 18 B	Re 11D	Asp 18B (O2) - 2.1<br>Asp 18B (O1) – 3.2<br>O-Re-O bite angle = 44°	45	26.3
D6	Asp 52A	Re 6D	Asp 52A (O2) – 2.3<br>Asp 52A (O1) – 3.4<br><br>Re 6D (Asp 52A) … Re 5D (Glu 35A) dist = 4.0	42	36.2
D14	Asp 52B	Re 14D	Asp 52B (O2) - 2.4<br>Asp 52B(O1) – 3.6<br><br>Re14D(Asp52B) … Re 13D(Glu35B) dist = 3.8	43	32.9
D2	Asp 119A<br><br>Gln 121A	Re 2D	Asp119A (OD2) – 2.5<br>Asp119A (OD1) – 3.2<br>Gln121A (NE2) – 1.9	40	30.2

D10	Asp 119B	Re 10D	Asp119B (OD2) – 2.6	45	42.4
D4	Leu 129A	Re 17D & Re 18D<br><br><br><br><br>Re 4D	Re 17D - Leu129A (O) = 2.7<br>Re 17 D - Leu129A (OXT) = 3.3<br><br>Re17D…Re18D = 1.8<br><br>Re 4D – Leu 129A (O) = 3.5<br>Re 4D – Leu 129A (OXT) = 2.9	50 & 45<br><br><br><br><br><br><br>16	24.8 & 21.0<br><br><br><br><br><br><br>22.1
D12	Leu 129B	Re 12D	Leu129B (O) – 2.3<br>Leu129B (OXT) – 3.1<br>O-Re-O bite angle = 43°<br>(2Fo-Fc density is better for Leu129B than Leu129A)	17	23.4
D5	Glu 35A	Re 5D	Glu35A (O) – 2.7	30	36.4
D13	Glu 35B	Re 13D	Glu35B (O) – 2.6	32	42.5
D7	Near Pro 70A	Re 7D	Pro70A (O) – 3.7	27	32.2
D15	Near Pro 70B	Re 15D	Pro70A (O) – 3.7	30	31.0
D8	In vicinity of Glu 7B, Arg 14A & His 15A	Re 8D	Glu 7B(O) - 3.2	30	28.0

D16	In vicinity of Glu 7A, Arg 14B & His 15B	Re 16D	Glu 7A(O) – 3.4	40	35.7

[a] In the case of anisotropic protein model refinement undertaken at a diffraction resolution worse than ~1.6 Angstrom the Calc DPI formula denominator value of (Number of observations - Number of refined parameters) can approach zero and the DPI estimate thereby becomes unstable. Therefore the distance values of our CuKα cannot reliably report ESD values and which are therefore not included in this table. Details regarding the 'DPI webserver' can be found in Kumar *et al*. *J Appl Cryst* 2015.

Table S4 Table of selected bond distances and angles found in the Diamond Light Source (λ = 0.9763 Å) diffraction dataset for the cases not discussed in the main text. Their occupancies and B factors are given in Table S1a above.

Chain	Residue	Bond distance (Å)	Bond angle (°)
B	Asp 119B	Re4D-OD2 = 2.53(9)<br>Re4D-OD1 = 3.38(9)	OD1-Re-OD2 = 40(2)
B	Asp 18B	Re5H-OD2 = 2.26(9)<br>Re5H-OD1 = 3.28(8)	OD1-Re-OD2 = 39(1)
B	Asp 52B	Re 9H-OD2 = 2.2(2)<br>Re9H…Re8H = 4.2(1)	
B	Glu 35B	Re8H-OE1 = 2.69(7)	

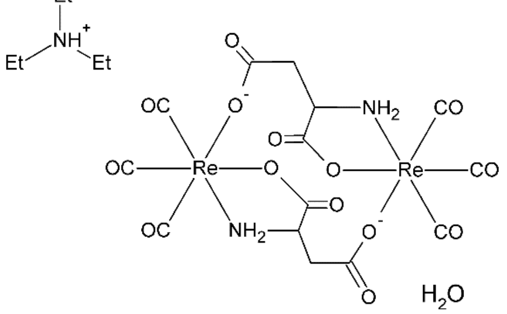

Figure S1 Representation of the unusual cyclic dimer CSD-REFCOD: UDENAU whereby one Re binds coordinates to two aspartic acid subunits.

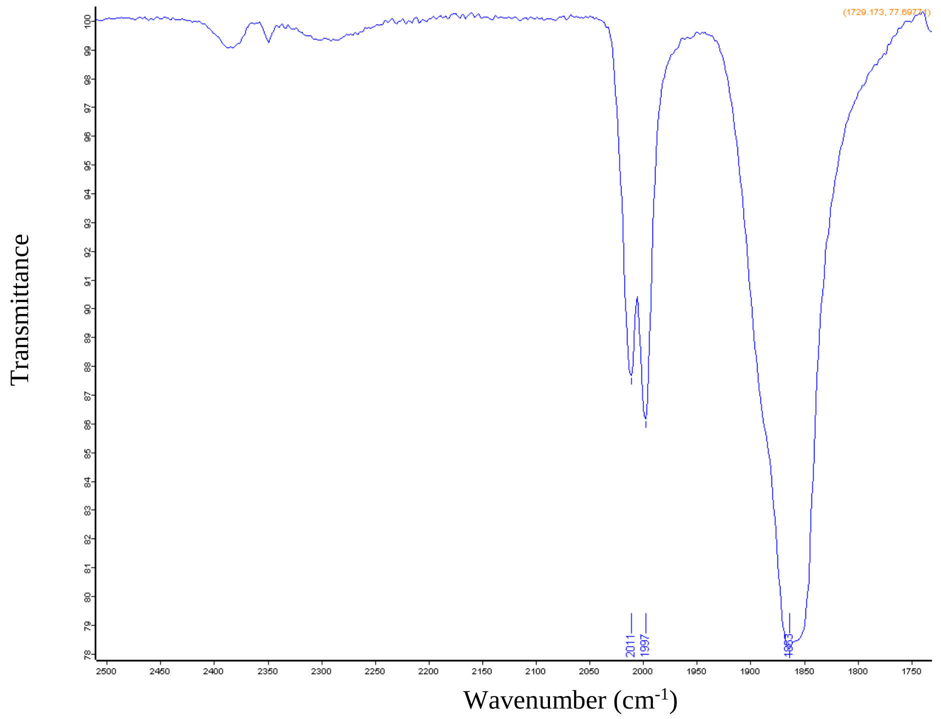

Figure S2 IR spectra of the carbonyl stretching region of *fac*-$[Re(CO)_3(H_2O)_n]^+$ ($n \leq 3$) bound to HEWL.
